# Co@Carbon and Co_**3**_O_4_@Carbon nanocomposites derived from a single MOF for supercapacitors

**DOI:** 10.1038/s41598-017-12733-5

**Published:** 2017-10-03

**Authors:** Engao Dai, Jiao Xu, Junjie Qiu, Shucheng Liu, Ping Chen, Yi Liu

**Affiliations:** School of Physical Sciences, Guizhou University, Guiyang, 550025 China

## Abstract

Developing a composite electrode containing both carbon and transition metal/metal oxide as the supercapacitor electrode can combine the merits and mitigate the shortcomings of both the components. Herein, we report a simple strategy to prepare the hybrid nanostructure of Co@Carbon and Co_3_O_4_@Carbon by pyrolysis a single MOFs precursor. Co-based MOFs (Co-BDC) nanosheets with morphology of regular parallelogram slice have been prepared by a bottom-up synthesis strategy. One-step pyrolysis of Co-BDC, produces a porous carbon layer incorporating well-dispersed Co and Co_3_O_4_ nanoparticles. The as-prepared cobalt-carbon composites exhibit the thin layer morphology and large specific surface area with hierarchical porosity. These features significantly improve the ion-accessible surface area for charge storage and shorten the ion transport length in thin dimension, thus contributing to a high specific capacitance. Improved capacitance performance was successfully realized for the asymmetric supercapacitors (ASCs) (Co@Carbon//Co_3_O_4_@Carbon), better than those of the symmetric supercapacitors (SSCs) based on Co@Carbon and Co_3_O_4_@Carbon materials (i.e., Co@Carbon//Co@Carbon and Co_3_O_4_@Carbon//Co_3_O_4_@Carbon). The working voltage of the ASCs can be extended to 1.5 V and show a remarkable high power capability in aqueous electrolyte. This work provides a controllable strategy for nanostructured carbon-metal and carbon-metal oxide composite electrodes from a single precursor.

## Introduction

The carbon-based electrical double-layer supercapacitors (EDLC) have excellent cyclic stability and long service lifetime since the electrode undergoes no chemical change during the charge/discharge processes^1,2^. However, the energy density of currently commercial carbon-based EDLC is much lower than that of an electrochemical battery. Such low energy-density cannot fulfill the need of energy storage devices for hybrid electric vehicles, wind-farms and solar power plants. Recently, 2-D carbon nanostructures combine the high surface area, high electronic conductivity and high mechanical strength, which are very attractive for flexible energy-storage devices and for improvement in the charge/discharge reaction kinetics of supercapacitor electrodes^3–7^. Graphene, graphene oxide (GO) or reduced graphene oxide (rGO) are the representatives of the 2-D carbon nanosheets. Graphene and GO exhibit great mechanical strength, excellent electronic conductivity as well as high specific surface area, which are promising candidates for supercapacitor electrodes. However, the synthesis of graphitic-ordered mesoporous carbon is still one of challenges. Heat treatment that requires high temperature over 2000 °C is a traditional way, yet the process is rather energy costly. Employing inorganic additives for catalytic graphitization of amorphous carbon at relatively low temperature (<1000 °C) is an alternative to the high temperature method. Some initial work has reported that metal salts containing Fe, Co, Ni, Ti, W, and Mn exhibit catalytic graphitization behavior at the low temperature^8–11^. When hybridized with other metal or metal oxide nanoparticles (NPs) to form carbon/metal/metal oxide composites, multi-functionality is achieved through the combination of carbon and metal or metal oxide^12–17^. In these composites, the carbon nanostructure serves as the physical support of metal/metal oxide particles and its structure determines the architecture of the whole composite. The high electronic conductivity of carbon nanostructures benefits to the rate capability and power density at a large charge/discharge current. The electro-activities of metal/metal oxide NPs contribute to high specific capacitance and high energy density of the composite electrodes. A synergistic effect could be expected and the materials cost can be reduced.

In most cases, metal cations deposited on carbonaceous materials were reduced chemically or physically to form carbon/metal/metal oxide nanocomposites. In these processes, heterogeneous dispersion and agglomeration of the particles are problematic. As a relatively new class of porous materials, metal-organic frameworks (MOFs), usually constructed from metal (clusters) and organic ligands with diversified and tailorable structures have been primarily demonstrated to be suitable templates/precursors to afford uniform metal (oxide) NPs distributed throughout porous carbon via pyrolysis, in which high porosity and long-range structural ordering could be partially preserved^18–25^. The agglomeration of metal (oxide) clusters would be limited due to the presence of the surrounding polymers, which would be stabilized and slowly carbonized to form the metal (oxide)-core/carbon-shell architectures^25–27^.

**Figure 1:**
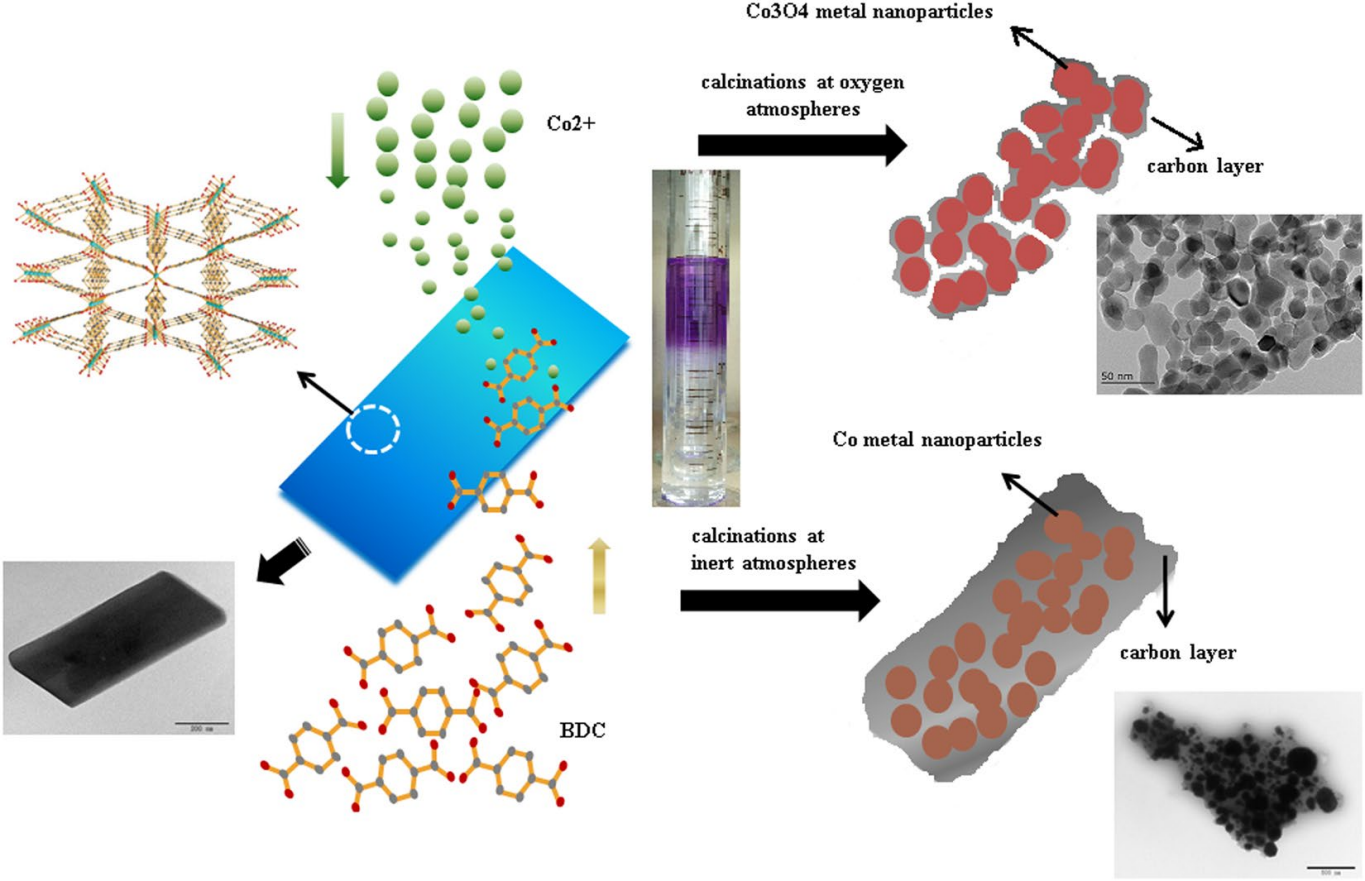
Synthetic scheme for the preparation of Co-BDC nanosheets and cobalt-carbon composites.

In the present work, we have constructed an elegant nanostructure in which cobalt NPs was over coated by ultrathin carbon layers by using Co-containing MOF (Co-BDC) as both self-sacrificing template and precursor. Bottom-up synthesis method is an interesting strategy to produce highly crystalline and intact MOF nanosheets^9^. The bottom-up synthesis of MOF nanosheets and conversion of cobalt-carbon nanocomposites was schematically illustrated in Fig. 1. A topmost solution of cobalt(II) acetate tetrahydrate and a bottom solution of 1,4-benzenedicarboxylic acid (BDCA), separated by an intermediate solvent layer (mixtures of N,N-dimethyl formamide and acetonitrile). Under static conditions, diffusion of Co^2+^ cations and BDCA linker precursors into the space segment causes a slow supply of the MOF nutrients to an intermediate region where the growth of MOF crystals occurs locally. Finally, the Co-BDC nanosheets were calcined in an inert atmosphere at 700 ^o^C to obtain Co@Carbon. Then Co-BDC was calcined in oxygen atmosphere at 400 ^o^C to obtain Co_3_O_4_@Carbon. The designed two hybrid electrode shows a special core-shell structure and porous feature, which present a high specific capacitance and good rate capability.

## Results and Discussion

**Figure 2:**
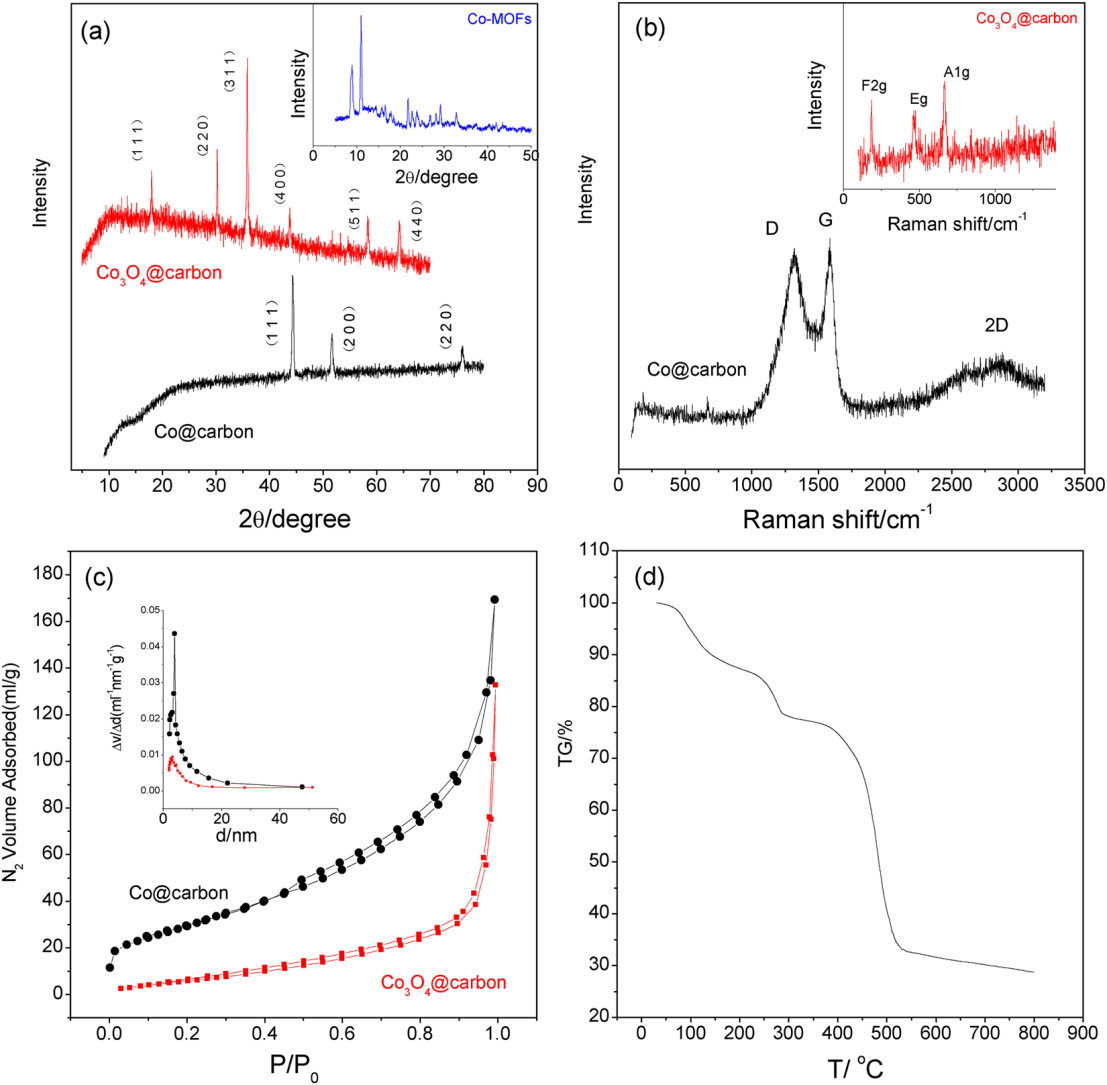
(**a**) XRD, (**b**) Raman spectra, (**c**) N_2_ adsorption and desorption isotherms, and (**d**) TG of samples.

Typical XRD patterns of Co@Carbon and Co_3_O_4_@Carbon are displayed in Fig. 2(a). The XRD patterns of the samples agree well with the cubic Co (JCPDS: 15–0806) and Co_3_O_4_ (JCPDS: 42–1467) phase with amorphous carbon bulge, respectively. No peaks from impurity phases can be detected, demonstrating high purity of hierarchical Co@Carbon and Co_3_O_4_@Carbon hybrid.

The Raman spectrum of Co@Carbon, as shown in Fig. 2(b), displayed the well documented D band at 1328 cm^−1^ and G band at 1591 cm^−1^ further confirming the existence of carbon in the hybrid. The D band can be assigned to typical disorder, while the G band is characteristic of grapheme^28^. The intensity ratio between the D and G bands (I_D_/I_G_ ~1.0) demonstrates that the carbon in the hierarchical Co@Carbon material is amorphous. The results prove that as-obtained composites have a large amount of void space for volume expansion and provide numerous electro-active sites for redox reactions. Obviously, the emergency of 2D band at 2645 cm^−1^ clearly indicates that the existence of ultrathin carbon layers in Co@Carbon hybrids^29,30^. For Co_3_O_4_@Carbon, the F_2g_, E_g_ and A_1g_ peak assigned to Co_3_O_4_ were observed in Raman spectrum (Inset of Fig. 2(b))^31^.

Nitrogen adsorption and desorption measurements of as synthesized Co@Carbon and Co_3_O_4_@Carbon were performed to obtain more information on the porous structure (Fig. 2(c)). The type IV isotherms can be assigned to type of H3 hysteresis loops, indicating the presence of mesoporous structure. The BET surface area of Co@Carbon and Co_3_O_4_@Carbon is about 109.6 m^2^ g^−1^ and 23.6 m^2^ g^−1^, respectively. Inset of Fig. 2(c) shows the pore-size distribution calculated by the Barrett-Joyner-Halenda (BJH) method. It shows an average pore-size value of about 9.6 nm for Co@Carbon and 34.8 nm for Co_3_O_4_@Carbon. We believe that the mesoporous structure is critical for facilitating the transfer of electrons and ions in the interface between the electrode and electrolyte and offers many active sites for fast electrochemical reactions. This may lead to a great enhancement of electrochemical properties.

**Figure 3:**
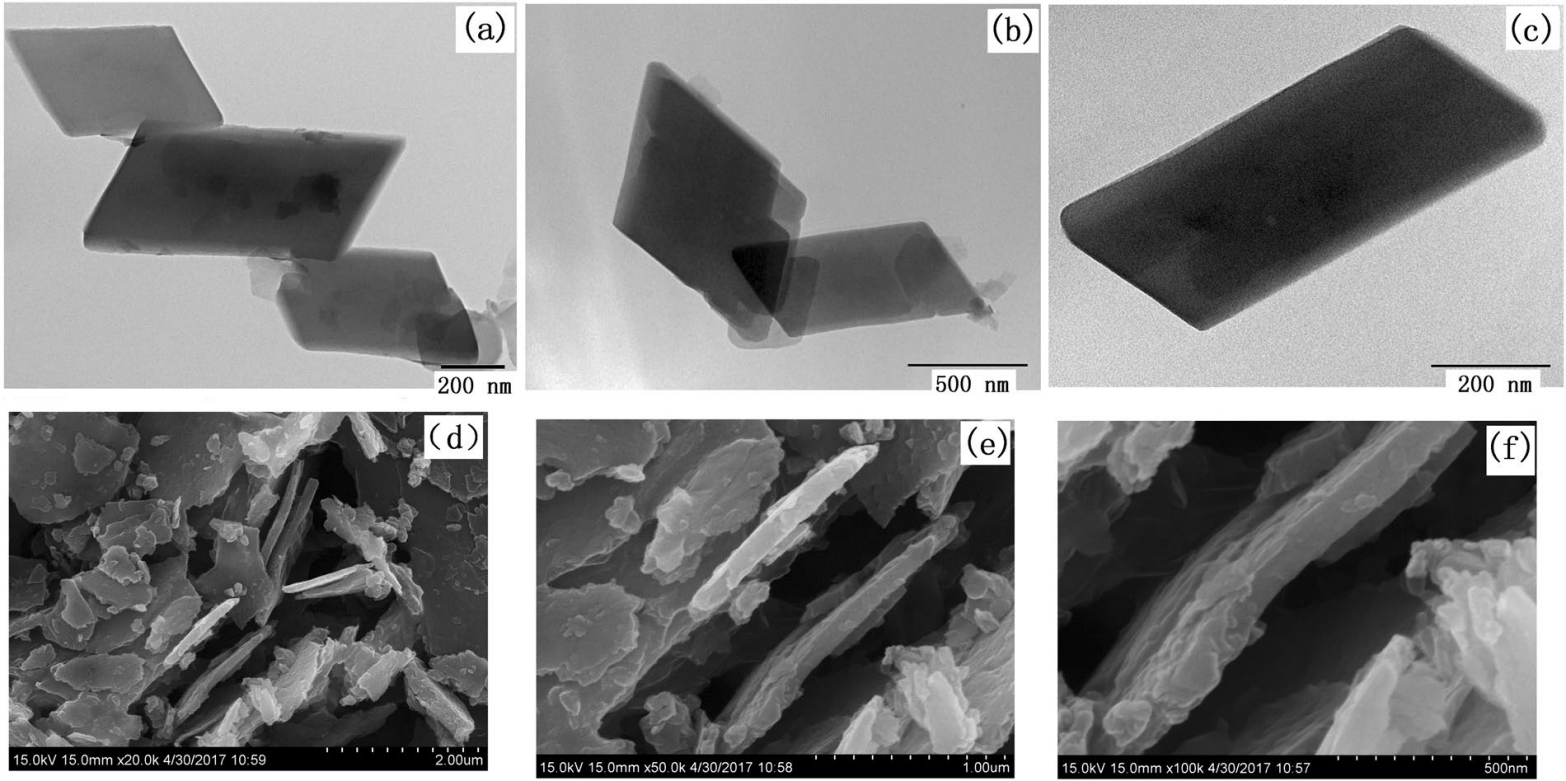
(**a**–**c**) TEM and (**d**–**f**) SEM of Co-BDC nanosheets.

Transmission electron microscopy (TEM) images of Co-BDC precursor are shown in Fig. 3(a–c) on different resolution scales. It was observed that the Co-BDC exhibits the morphology of regular parallelogram slice with width and lengths up to several micrometers. Scanning electron microscopy (SEM) was employed to investigate the as-collected samples as shown in Fig. 3(d–f). The corresponding magnified SEM images indicate that the Co-BDC exhibits uniform sheet-shaped morphology with layer thickness down to 100 nm.

**Figure 4:**
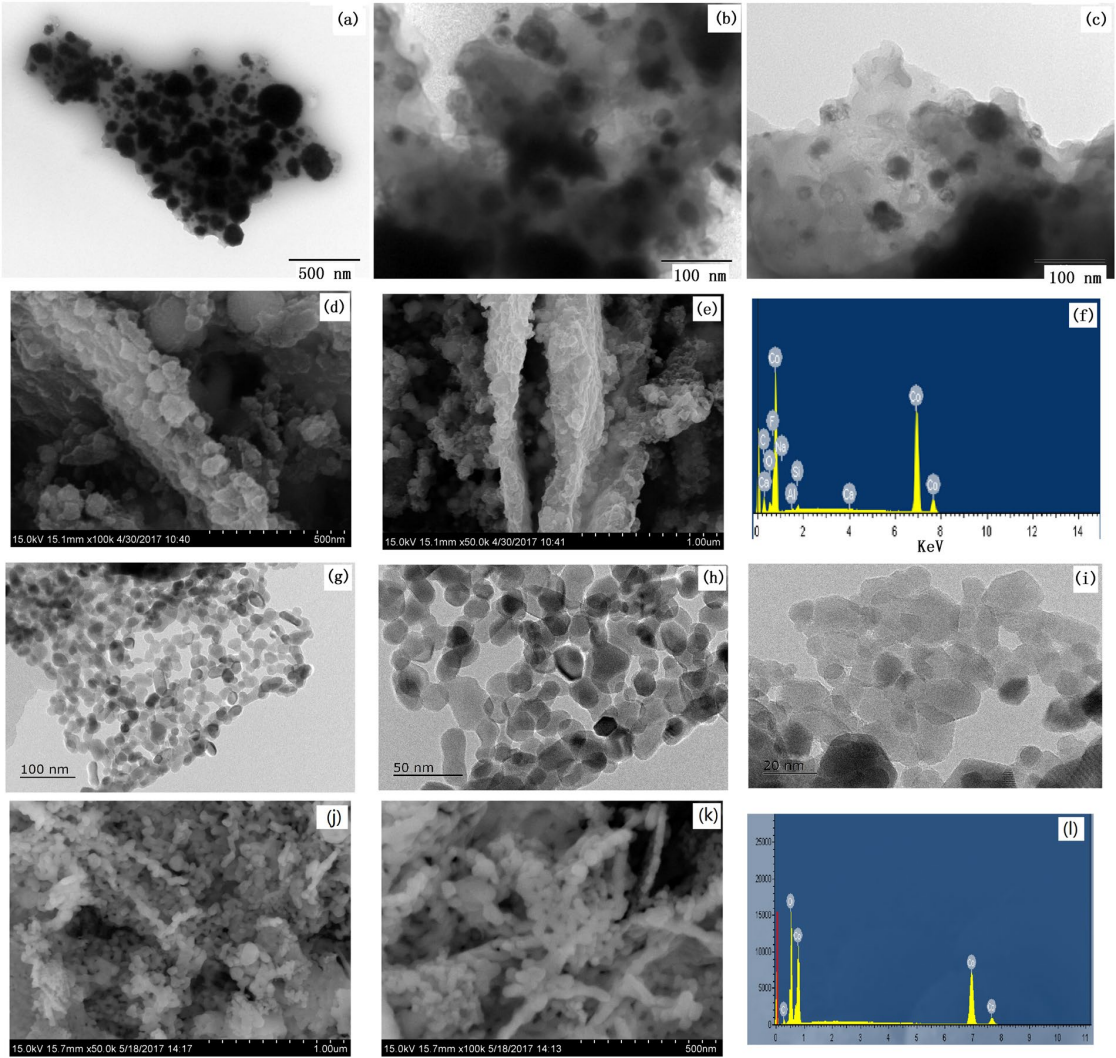
(**a**–**c**) TEM, (**d**,**e**) SEM, and (**f**) EDS of Co@Carbon; (**g**–**i**) TEM, (**j**,**k**) SEM, and (**l**) EDS of Co_3_O_4_@carbon.

**Figure 5:**
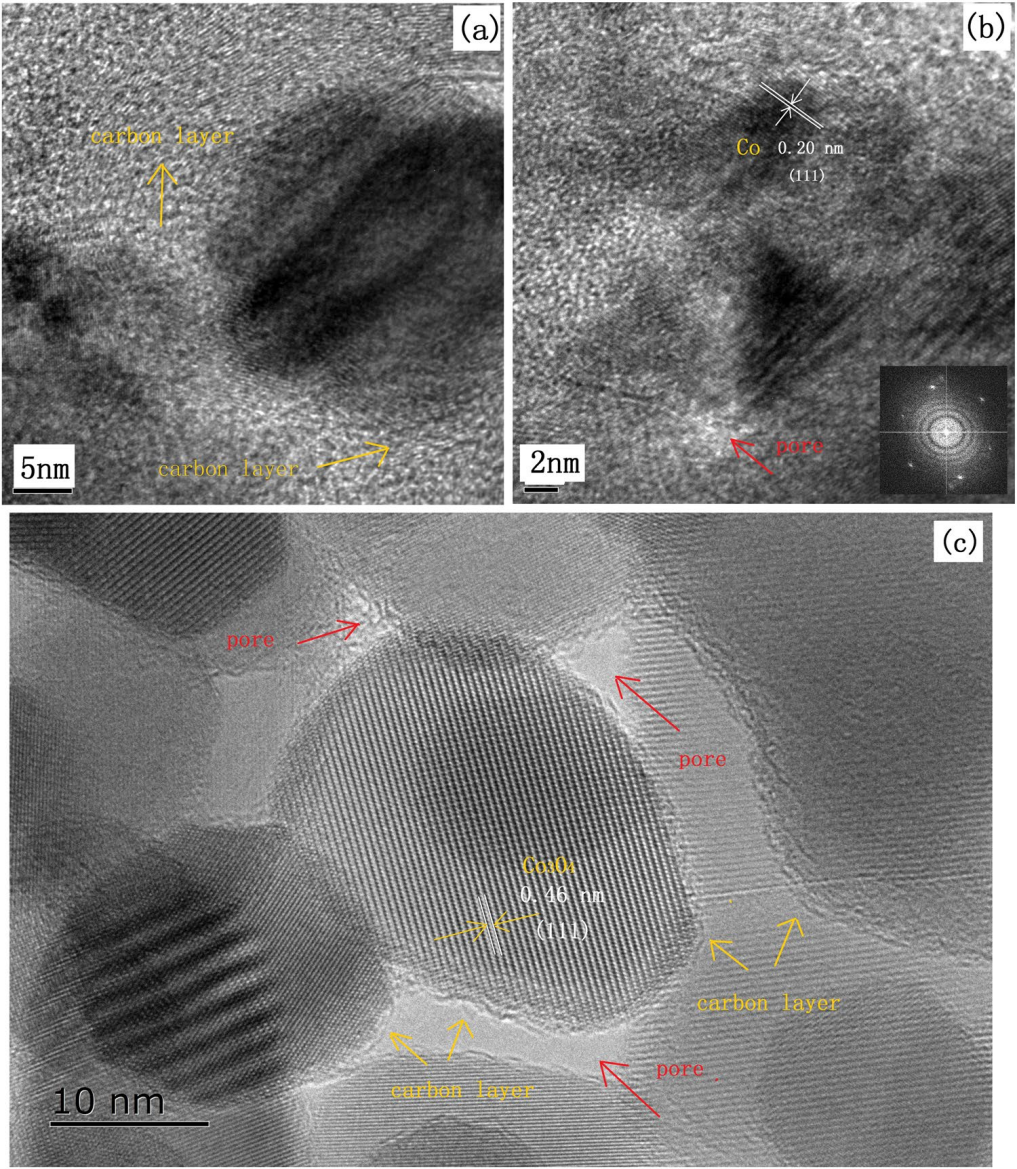
HRTEM of (**a**,**b**) Co@Carbon and (**c**) Co_3_O_4_@Carbon.

TEM was also used to collect more information of Co@Carbon as displayed in Fig. 4(a–c). From images, it is detected that the Co nanoparticles with around 10 to 30 nm in diameter are monodispersed within the thin carbon layer. As shown in the SEM (Fig. 4(d–f)), the Co@Carbon was composed of thin carbon nanosheets with about 150 nm thicknesses, and the coarse surface with Co nanoparticles. Heat treatment of Co-MOFs in O_2_ resulted in nanometer-sized spherical cobalt-carbon composites Co_3_O_4_@Carbon (Fig. 4(g–k)). HRTEM characterization disclosed that these small particles were carbon-shell/cobalt-core hybrids (Fig. 5(c)). The diameter of the Co_3_O_4_ particles ranged from 10 to 30 nm. The EDS of materials were shows in Fig. 4(f,l) and the elemental composition of materials analyzed by EDS were presented in Tables S1 and S2. High-resolution TEM (HRTEM) (Fig. 5) analysis showed that the cobalt particles were composed of single-crystal structures and the carbon layers covered the particle surface. The HRTEM image reveals a fringe spacing of around 0.20 nm and 0.46 nm, corresponding to the (111) plane of cubic Co and Co_3_O_4_.

These TEM and SEM images above clearly bring out some peculiar features for both cobalt-carbon composites, are to be noted as (a) typical few layer carbon character with significant layered nature, (b) uniform cobalt nanoparticle loading across the extended sheets, and (c) hierarchical porosity ranging from uniformly dispersed tiny nm scale pores to scattered mesopores. According to thermogravimetric analysis (TGA) in N_2_ atmosphere, Co-MOFs start to decompose at 700 °C (Fig. 2(d)). One can speculate that *in situ* carbonization of Co-MOFs would lead to the formation of cobalt clusters surrounded by ligands framework. The agglomeration of cobalt clusters would be limited due to the presence of the surrounding ligands, which would be stabilized and slowly carbonized to form the cobalt-core/carbon-shell architectures. We believe that the *in situ* process is beneficial for high interfacial interaction between the cobalt and carbon in the samples. Thus, the intimate combination of cobalt with electronically conducting carbon allows for rapid and efficient charge transport, which can lead to great improvement of electrochemical properties.

**Figure 6:**
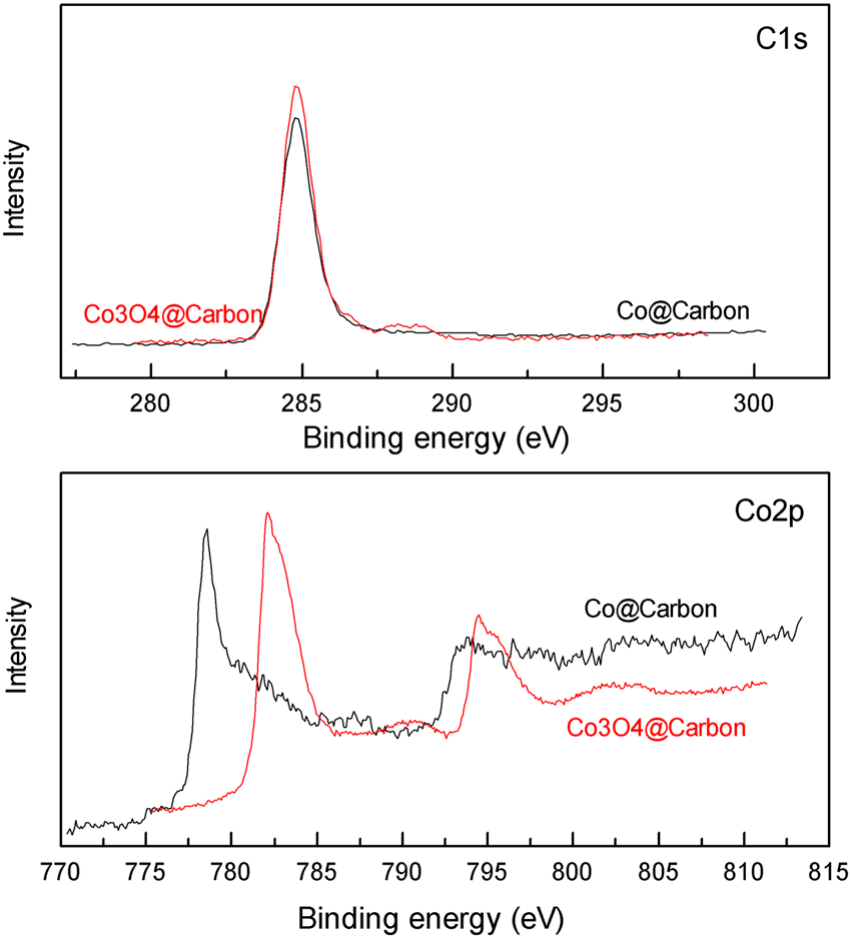
XPS of cobalt-carbon composites.

The X-ray photoelectron spectroscopy (XPS) spectra of as-obtained cobalt-carbon nanocomposites are presented in Fig. 6. The peak centered at 284.6 eV was observed in both samples, which is referred to characteristic peak of C1s. For Co@Carbon, the peak located at 778.5 eV is assigned to the characteristic peak of Co_2p3/2_, suggesting the presence of zero valence Co^32^. The presence of zero valences Co and carbon confirms the successful deposition of Co@Carbon material. XPS revealed Co/C atomic ratio of about 6% in Co@Carbon hybrid. For Co_3_O_4_@Carbon, the binding energy peaks at 779.8 eV, which can be attributed to Co^2+^ and Co^3+^ states^33,34^, suggesting the formation of Co_3_O_4_@Carbon. What’s more, the Co/C atomic ratio determined from XPS measurement is much high than 50%. It is probably because most of the carbon is vaporized during sintered in the oxygen atmosphere.

**Figure 7:**
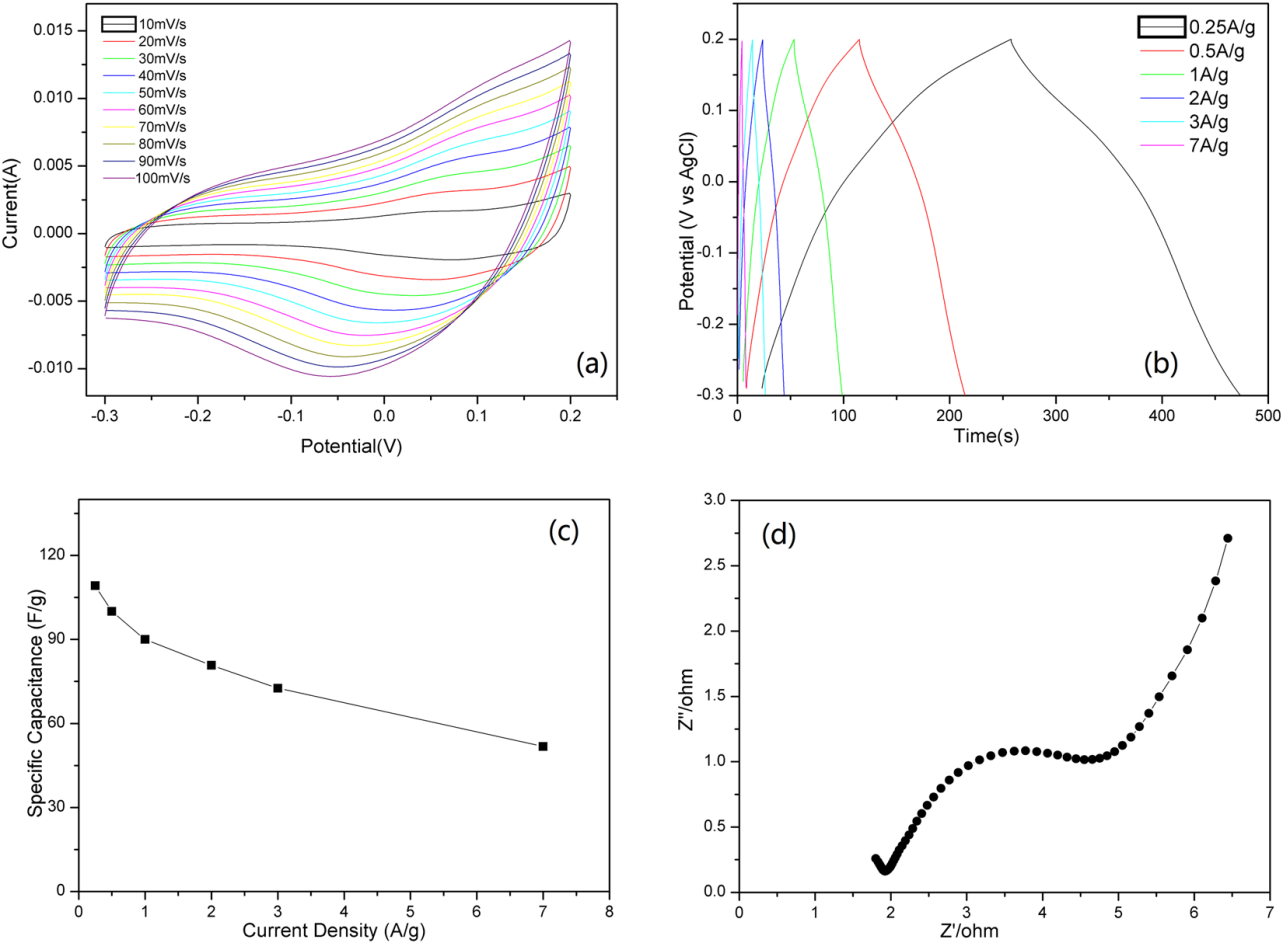
(**a**) Cyclic voltammetry curves, (**b**) galvanostatic charge-discharge curves, (**c**) Specific capacitance at different current density, (**d**) Nyquist plots of the Co@Carbon electrode.

The electrochemical performance of Co@Carbon and Co_3_O_4_@Carbon was evaluated in a three-electrode configuration with a 6 M KOH electrolyte. The representative cyclic voltammetry (CV) curves of Co@Carbon are shown in Fig. 7(a) with the scan rates varying from 10 to 100 mV s^−1^. These curves show the combination of both pseudo capacitive and an electric double layer capacitive behavior between −0.3 and 0.2 V vs. Ag/AgCl electrode, indicating the good capacitive properties. An obvious redox peak was observed, which was attributed to the reversible oxidation state change of Co element between Co^2+^ and Co^3+^. With the increase of scan rate, the shape of CV curves did not change too much and the redox peaks well remained, indicating the high rate capability of the Co@Carbon electrode. The Co@Carbon electrode was further tested by galvanostatic charge/discharge measurements (Fig. 7(b)). The charge/discharge curves at different current densities exhibited an almost symmetric shape with a small voltage drop, indicating the Co@Carbon electrode has small internal resistance and excellent capacitive property. The capacitance values are estimated to be 109, 100, 90, 81, 73 and 52 F g^−1^, respectively, at current densities of 0.25, 0.5, 1, 2, 3 and 7 A g^−1^. The electrochemical performances of Co@Carbon were further investigated by electrochemical impedance spectroscopy (EIS) from 10^5^ to 10^−2^ Hz, and the Nyquist plots are drawn in Fig. 7(d). In the high frequency region, the intercept on the real axis can reflect the resistance of the electrolyte (R_s_). Rs of material are about 2.0 Ω. The semicircular pattern in the middle frequency region is related with the resistance of charge transfer (R_ct_). The R_ct_ value of material are much lower (~4.0 Ω) due to its nanosheet structure with high mesoporous surface area. It is easy for electrolyte to soak in contact with electrode material. The slope line in the low frequency region is related with Warburg impedance of ion diffusion/transportation to the electrode surface. The steeper the slope line is, the faster ion diffusion is. So, Co@Carbon displayed favorable ion diffusion and showed excellent supercapacitor performance especially at a high current density.

**Figure 8:**
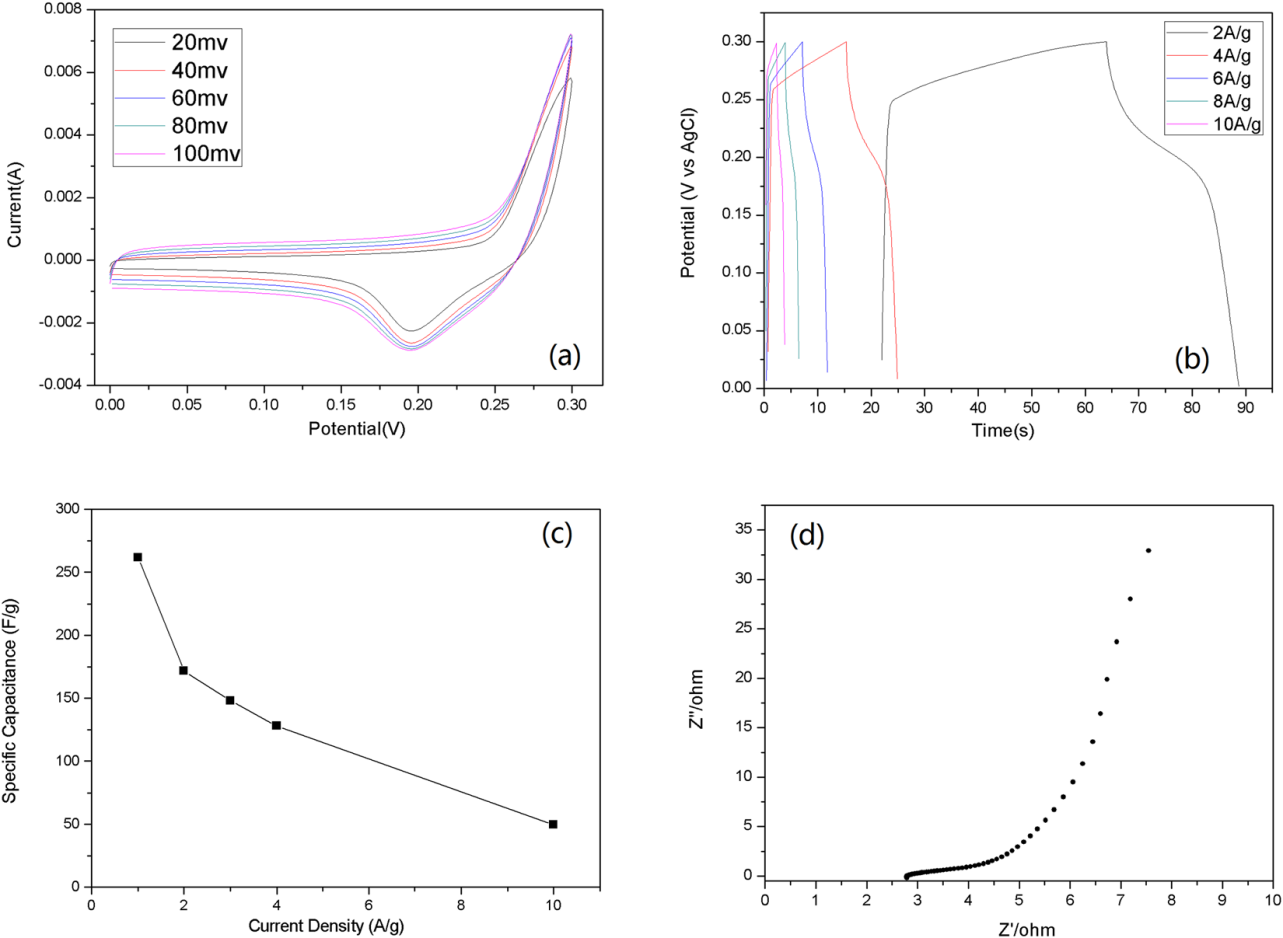
(**a**) Cyclic voltammetry curves, (**b**) galvanostatic charge-discharge curves, (**c**) Specific capacitance at different current density, (**d**) Nyquist plots of the Co_3_O_4_@Carbon electrode.

The representative CV curves of Co_3_O_4_@Carbon are shown in Fig. 8(a) at different scan rates. The CV shows a typical pseudo capacitive behavior with an obvious redox peak, which was attributed to the reversible conversion between Co^2+^ and Co^3+^. The discharge behaviors of Co_3_O_4_@Carbon were examined by GCD in the potential range from 0 to 0.3 V at current densities of 2~10 A g^−1^. The GCD curves (Fig. 8(b)) of Co_3_O_4_@Carbon exhibited a more obvious voltage platform due to the redox reaction. The specific capacitance was calculated to be 261, 171, 148, 128 and 50 F g^−1^ at the current density of 1, 2, 3, 4 and 10 Ag^−1^. The capacitance value of Co_3_O_4_@Carbon was about 3 times higher than that of Co@Carbon at 1 Ag^−1^. Faradaic capacitance of Co_3_O_4_ at around 0.19 V associated with the conversion from Co^3+^ to Co^2+^ was mainly responsible for the high capacitance. Moreover, The R_ct_ values of Co_3_O_4_@Carbon (∼2 Ω) are lower than that of Co@Carbon (∼4 Ω). The results indicate the coating of carbon thin layer on Co_3_O_4_ played an essential role in the enhanced performance of the composite electrode, which could be attributed to the unique structure of Co_3_O_4_@Carbon and the synergetic effect between carbon and Co_3_O_4_. In this special structure, the carbon thin film coated on Co_3_O_4_ and interconnected with each other to form conductive networks, promoting electron transfer. Moreover, the pore between Co_3_O_4_@Carbon nanostructures could also serve as the electrolyte ion reservoirs^11^, which not only ensured a close contact between the electrode material and the electrolyte, but also preserved a stable supply of electrolyte ions, leading to rapid ion transport. Furthermore, the incorporation of carbon led to the higher surface area of the Co_3_O_4_@Carbon composite and a smaller size of the Co_3_O_4_ nanomaterial, which not only shortened the ion diffusion length, but also provided more active surface area for the redox reaction.

**Figure 9:**
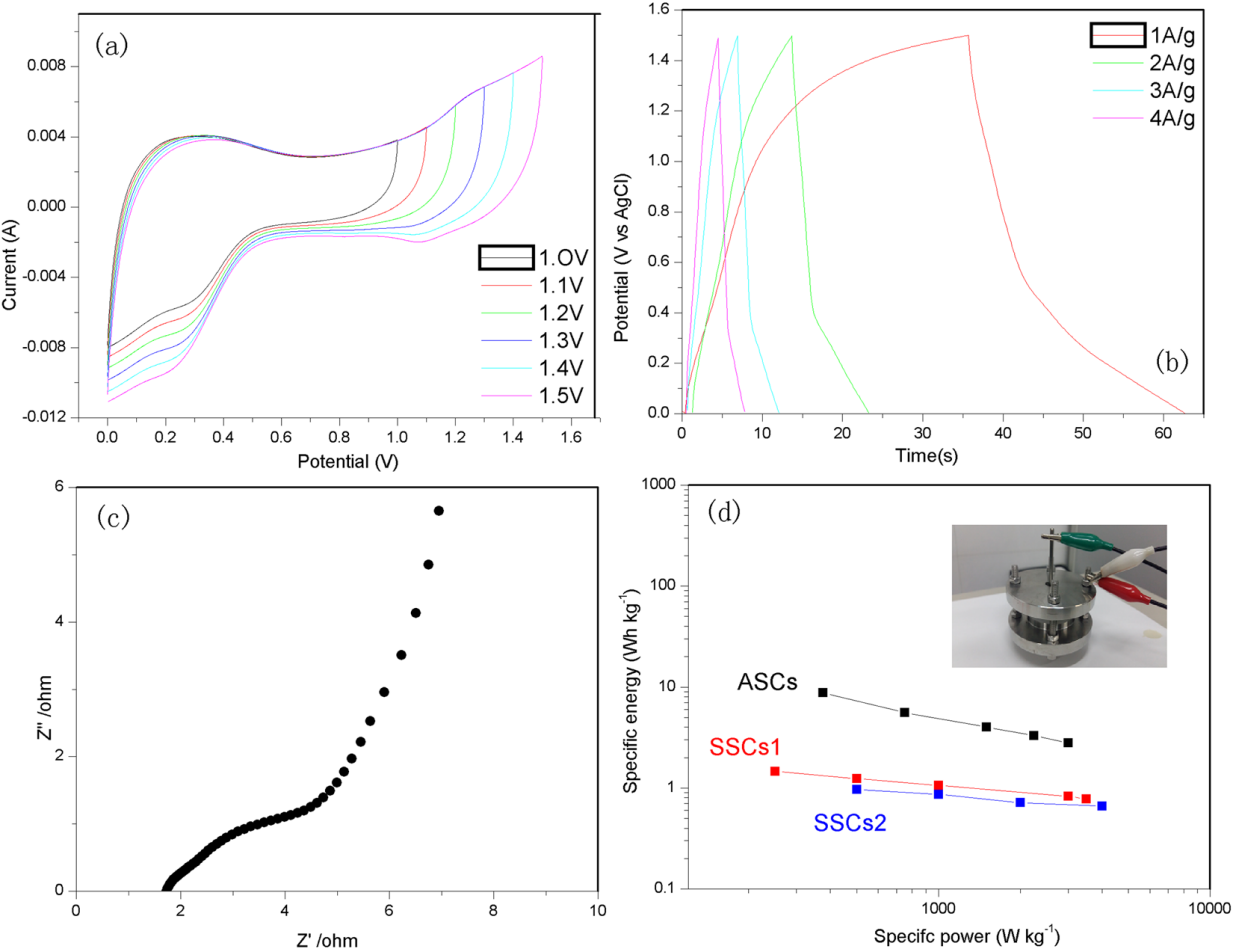
(**a**) Cyclic voltammetry curves at scan rates of 100mVs^−1^, (**b**) galvanostatic charge-discharge curves, (**c**) Nyquist plots, and (**d**) Ragone plots of the ASCs (Co@Carbon//Co_3_O_4_@Carbon), SSCs1(Co@Carbon//Co@Carbon) and SSCs2(Co_3_O_4_@Carbon//Co_3_O_4_@Carbon).

**Figure 10:**
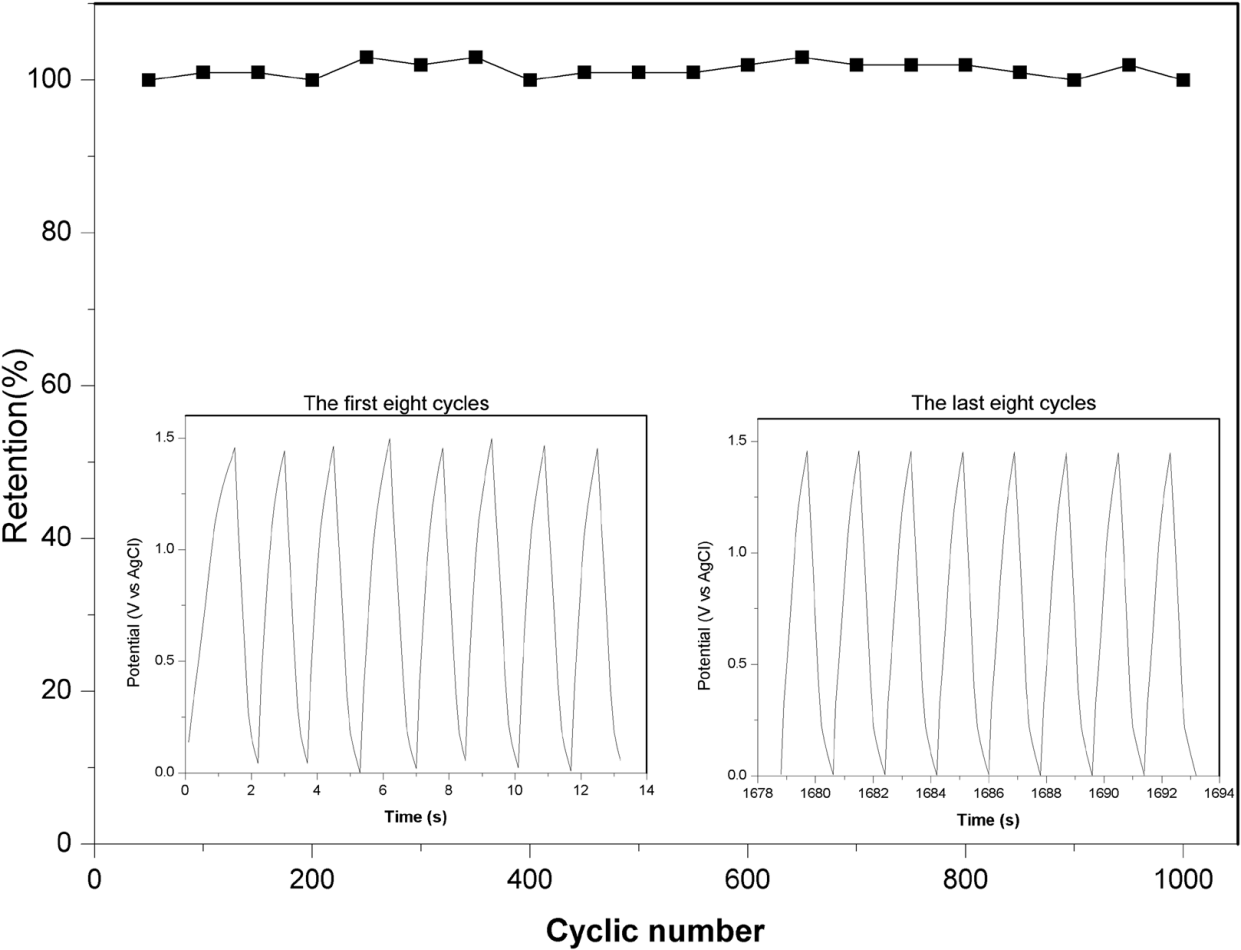
Cycle performance of Co@Carbon//Co_3_O_4_@Carbon ASCs at a current density of 10 A g^−1^.

In order to explore the electrochemical performance toward practical application of the materials, asymmetric supercapacitors (ASCs) was fabricated by utilizing the as-synthesized Co_3_O_4_@Carbon as the positive electrode and Co@Carbon as the negative electrode in 6 M KOH. To fabricate ASCs, The mass ratio of the negative electrode to the positive electrode was decided based on charge balance theory. It was observed that the CV curves (Fig. 9(a)) of ASCs are symmetric in shape even though the potential window is extended up to high working voltages, indicating ideal capacitive properties with good reversibility. The working voltage of the asymmetric supercapacitors thus can be extended to 1.5 V, indicating the potential of assembled system in practical application. The charge-discharge performance of the supercapacitors is also demonstrated by the galvanostatic charge-discharge curves presented in Fig. 9(b), which shows nearly symmetrical liner charge and discharge characteristics with no obvious internal voltage drops at different current densities, suggesting a highly reversible charge-discharge behavior. The specific capacitance was calculated from the GCD curves based on total mass loading of the active material of the two electrodes. The specific capacitance value calculated at a current density of 1 A g^−1^ was 17.9 F g^−1^, which only decreased to 8.9 F g^−1^, even at the high current density of 4 A g^−1^. The Nyquist plots (Fig. 9(c)) of ASCs show a lower R_ct_ (~4 Ω) and steeper slop at low frequency range, indicating fast of ion diffusion. To evaluate the cycle behavior of the as-fabricated ASCs, 1000 charge-discharge cycles were run at a current density of 10 A g^−1^. As shown in Fig. 10, during the 1000 cycles, the specific capacitances of the electrode almost maintain the initial value, implying that the composite is a stable electrode material during the cycling test. The power density and energy density are generally used as important parameters to characterize the performance of supercapacitor devices. Figure 9(d) gives the Ragone plot of the fabricated asymmetric supercapacitors for energy and power densities. The asymmetric supercapacitors achieve a high energy density of 8.8 Wh kg^−1^at a power density of 375 W kg^−1^. The specific energy and specific power change with the applied current density are summarized in Table 1. The values obtained for specific energy and specific power for the ASCs show improved performance.

**Table 1:** Various performance parameters for Co@Carbon//Co_3_O_4_@Carbon ASCs.

Potential range 0–1.5V				
Current density (A g^−1^)	Discharge time (s)	Specific capacitance (F g^−1^)	Specific energy (Wh Kg^−1^)	Specific Power (W Kg^−1^)
0.5	84.6	28.2	8.8	375
1	26.9	17.9	5.6	750
2	9.6	12.8	4.0	1500
3	5.3	10.6	3.3	2250
4	3.35	8.9	2.8	3000

To further demonstrate the advantage of ASCs, here, we fabricated two types of symmetric supercapacitors (SSCs) (Co@Carbon//Co@Carbon and Co_3_O_4_@Carbon//Co_3_O_4_@Carbon). It was found that the SSCs based on Co@Carbon//Co@Carbon exhibits only maximal specific energy of 1.24 Wh kg^−1^ at specific power of 500 Wkg^−1^, while the other SSCs based on Co_3_O_4_@Carbon//Co_3_O_4_@Carbon exhibits maximal specific energy of 0.97 Wh kg^−1^ at specific power of 500 W kg^−1^. The specific energy and specific power of two SSCs change with the applied current density as summarized in Tables S3 and S4. It was observed that the values of specific energy obtained for the ASCs are almost seven times higher than those for the SSCs. We think that the specially designed ASCs will link the advantages of these two materials to provide high specific energy and specific power. The presence of pseudocapacitive material Co_3_O_4_ helps in attaining higher current sweeps, meanwhile, the porous carbon is mainly responsible for providing a stable and wide potential window, which makes a major contribution to the high performance of the ASCs.

In summary, porous metal/carbon and carbon/metal oxide with well-controlled pore structures have been successfully prepared by a single MOFs-templating approach. We present a bottom-up synthesis strategy for preparation of Co-MOFs nanosheets with micrometre lateral dimensions and nanometre thickness. Cobalt/Carbon hybrids (Co@Carbon and Co_3_O_4_@Carbon) with high surfaces were obtained by one-step heat treatment of Co-MOFs. Improved capacitance performance was successfully realized for the ASCs utilizing the as-synthesized Co_3_O_4_@Carbon as the positive electrode and Co@Carbon as the negative electrode in aqueous electrolyte. This specially designed ASCs link the advantages of these two materials to provide high specific energy and specific power. The results presented here are of technological interest as this carbon/metal (oxide) composite are promising candidates for supercapacitors.

## Methods

### Material preparation

Cobalt 1,4-benzenedicarboxylate (Co-BDC) was synthesized by using Co^2+^ as metal cation and 1,4-benzenedicarboxylate (H_2_BDC) as organic ligand. A linker solution composed of 10 mg of H_2_BDC dissolved in a mixture of 2 mL of N,N-Dimethylformamide (DMF) and 1 mL of CH_3_CN was employed as bottom liquid layer, a mixture of 1 mL of DMF and 1 mL of CH_3_CN was the spacer layer, while a solution of 10 mg Co(CH_3_COO)_2_·4H_2_O in 1 mL of DMF and 2 mL of CH_3_CN was the top, metal-containing layer. Synthesis took place at 35 °C for 24 hours under static conditions. Finally, the solid product was recovered by centrifugation and thoroughly washed consecutively three times with DMF (1ml each step). And then placed in 80 °C thermostat box drying 2h. For the synthesis of Co@Carbon nanosheets, Co-BDC was placed in a tube furnace under N_2_ gas flow at 700 °C for 2 h with a heating rate of 3 °C/min. And for the synthesis of Co_3_O_4_@Carbon, Co-BDC was placed in a tube furnace under O_2_ gas flow at 400 °C for 1 h with a heating rate of 3 °C/min.

### Measurement

The crystal phase of all samples was characterized by powder X-ray diffraction (PANalytical X’Pert Powder diffractometer) with CuKa radiation. The morphology and microstructure of synthesized materials were characterized using a transmission electron microscope (TEM, Tecnai G2 F20). Nitrogen adsorption/desorption isotherms were measured at 77 K using surface area and pore size analyzer (3H-2000PS4). Raman spectra were recorded using a Renishaw inViaI instrument with a 633 nm laser.

The electrochemical measurements using a three-electrode electrochemical system, a 6 M KOH aqueous solutions as the electrolyte, Pt plate acted as the counter electrode and Ag/AgCl electrode served as the reference electrode. The working electrode consisted of active material, conductive graphite and PTFE with the mass ration of 8:1:1. The mixture of pulp was pasted onto Ni foam and vacuum dried 10 h.

Cyclic voltammetry and galvanostatic charge-discharge investigation were implemented using a CHI660E electrochemical workstation (ChenHua, Shanghai). The specific capacitance was calculated from the galvanostatic chargedischarge curves using the following equation:

1 $${\rm{C}}=\frac{{\rm{I}}\times {\rm{\Delta }}{\rm{t}}}{{\rm{m}}\times {\rm{\Delta }}{\rm{V}}}$$

where I is charge-discharge current at a discharge time ∆t (s), ∆V is dropout voltage, and m is the mass of active electrode materials.

For the fabrication of symmetric supercapacitors, the previous working electrode served as the positive electrode and the negative electrode. The two electrodes and a separator were combined with 6 M KOH as the electrolyte to assemble the full cell. The energy density E (Wh Kg^−1^) was used the following equation:

2 $${\rm{E}}=(0.5{{\rm{CV}}}^{2})/3.6$$

C (F g^−1^) is specific capacitance of capacitor, V is potential range. The power density P (WKg^−1^) was calculated by following equation:

3 $${\rm{P}}=3600{\rm{E}}/{\rm{t}}$$

where E (Wh Kg^−1^) is energy density, t(s) is elapsed time during discharge period.

## Electronic supplementary material

Supplementary Information
