# Supplementary material for: Co@Carbon and Co3O4@Carbon nanocomposites derived from a single MOF for supercapacitors
Source: Sci Rep. 2017 Oct 3;7:12588. doi: 10.1038/s41598-017-12733-5 (PMC5626685; doi:10.1038/s41598-017-12733-5)
Supplement: Supplementary file 1 — Supplementary Information [file 41598_2017_12733_MOESM1_ESM.pdf]

Supplementary Information

Co@Carbon and Co_3O_4@Carbon nanocomposites derived from a single MOF for supercapacitors

Engao Dai, Jiao Xu, Junjie Qiu, Shucheng Liu, Ping Chen, Yi Liu⁎

School of Physical Sciences, Guizhou University, Guiyang 550025, China

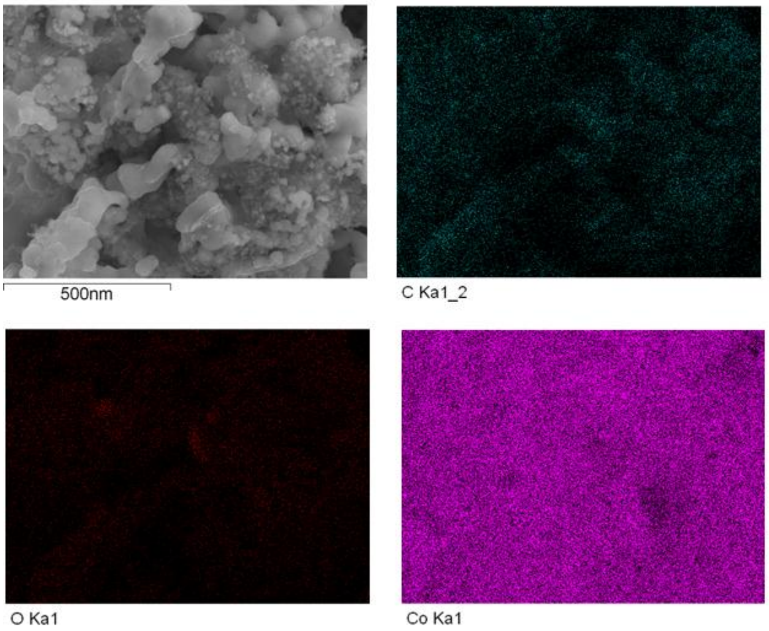

Fig. S1 EDS mappings of Co@Carbon

⁎ Corresponding author.
E-mail address: yliu9@gzu.edu.cn (Yi Liu)

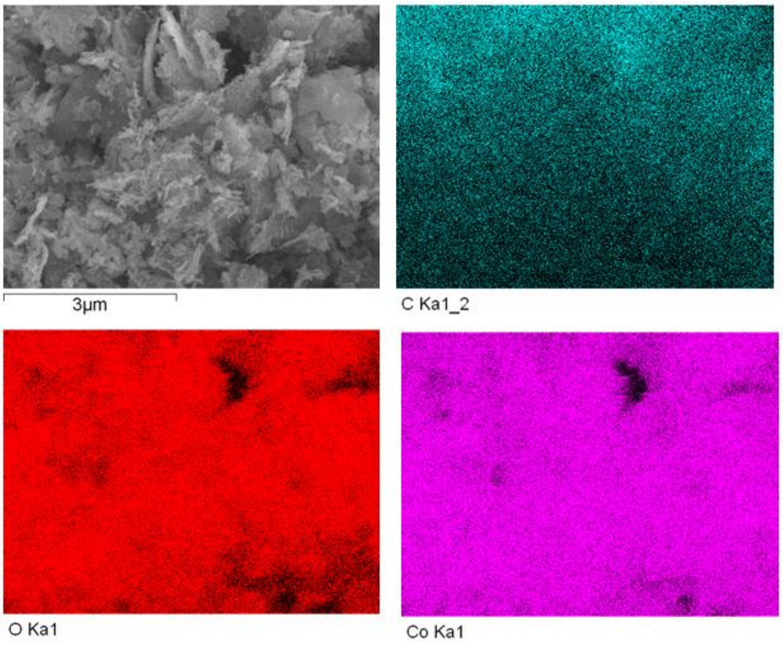

Fig. S2 EDS mappings of Co_3O_4@Carbon

Table S1. The chemical compositions of Co@Carbon detected by SEM-EDS

elements	mass %	atom%
C_K	9.17	31.59
O_K	1.53	3.96
Co_K	87.61	61.52

Table S2. The chemical compositions of Co_3O_4@Carbon detected by SEM-EDS

elements	mass%	atom%
C_K	3.36	8.78
O_K	27.72	54.46
Co_K	68.92	36.76

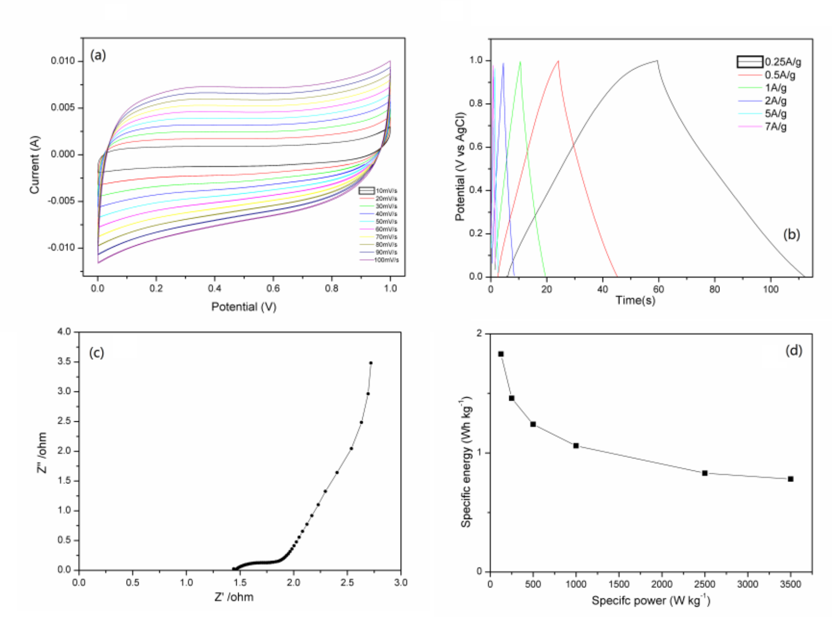

Fig. S3 Cyclic voltammetry curves (a), galvanostatic charge-discharge curves (b), Nyquist plots (c) and Ragone plots of the Co@Carbon //Co@Carbon SSCs

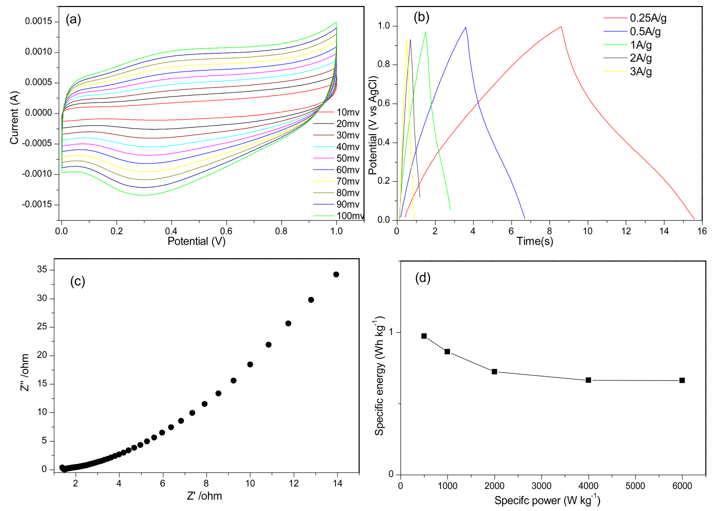

Fig. S4 Cyclic voltammetry curves (a), galvanostatic charge-discharge curves (b), Nyquist plots (c) and Ragone plots of Co_3O_4@Carbon//Co_3O_4@Carbon SSCs

TableS3. Various performance parameters for Co@Carbon // Co@Carbon SSCs

Current density (A g^{-1})	Discharge time(s)	Specific capaciance(F g^{-1})	Specific energy(Wh Kg^{-1})	Specific Power(W Kg^{-1})
0.25	52.7	13.2	1.83	125
0.5	21.0	10.5	1.46	250
1	8.9	8.9	1.24	500
2	3.8	7.6	1.06	1000
5	1.2	6.0	0.83	3000
7	0.8	5.6	0.78	3500

TableS4. Various performance parameters for Co_3O_4@Carbon // Co_3O_4@Carbon SSCs

Current density (A g^{-1})	Discharge time(s)	Specific capaciance(F g^{-1})	Specific energy(Wh Kg^{-1})	Specific Power(W Kg^{-1})
0.25	7.01	1.75	0.97	500
0.5	3.11	1.56	0.86	1000
1	1.3	1.3	0.72	2000
2	0.6	1.19	0.66	4000
3	0.4	1.19	0.66	6000

Table S5. Comparison of supercapacitor performance of Co-based nanomaterials.

Electrode materials	Cs	Rate performance	Cycling stability	Reference
**Co_3O_4@carbon**	**261 F g^{-1} at 1 A g^{-1} in 6 M KOH**	**Cs retention of 57% at 3A g^{-1}**	**~100% after 1000 Cycles in ASCs**	**This work**
Co_3O_4 nanostructures	208 F g^{-1} at 0.5 A g^{-1} in 3 M KOH	Cs retention of 36% at 6 A g^{-1}	97.5% after 1000 cycles	Dalton, Trans, 2012, 41, 5862.
Co_3O_4 microspheres	102 F g^{-1} at 3mVs^{-1} in 4 M KOH	--	74% after 500 cycles	Current. Appl. Phys, 2010, 10, 1422.
need-like Co_3O_4	111 F g^{-1} at 1 A g^{-1} in 2 M KOH	--	88.2% after 1000 cycles	J. Mater. Chem., 2010, 20, 7015
Co_3O_4 nanosheets	92 Fg^{-1} at 5 mAcm^{-2} in in 3 M KOH	Cs retention of 93% at 20 mAcm^{-2}	--	Chem. Eur. J., 2009, 15, 5320
Co_3O_4 nanowires	163 F g^{-1} at 1 A g^{-1} in 3 M KOH	Cs retention of 74% at 3A g^{-1}	98% after 1000 cycles	Acta Phys. -Chim. Sin., 2012,28 (02), 387.
Co_3O_4 derived from Co-MOF	126 F g^{-1} at 1A g^{-1}in 2 M KOH	Cs retention of 94% at 3A g^{-1}-	~100% after 3400 cycles	J. Mater. Chem. A, 2013,1, 7235
Co_3O_4 nano/micro superstructures from MOF	208 F g^{-1} at 1Ag^{-1} in 6 M KOH	Cs retention of 50% at 3A g^{-1}	97 % after 1000 cycles	Int. J. Electrochem. Sci., 2011,6,2943
Co_3O_4 prepared from MOF	241Fg^{-1} at 200 mVs^{-1} in 6 M KOH	Cs retention of 52% at 200 mVs^{-1}	--	Acs Nano , 2015, 9 (6),6288
